# Using a concurrent challenge with porcine circovirus 2 and porcine reproductive and respiratory syndrome virus to compare swine vaccination programs

**DOI:** 10.1038/s41598-022-19529-2

**Published:** 2022-09-15

**Authors:** Adthakorn Madapong, Kepalee Saeng-chuto, Angkana Tantituvanont, Dachrit Nilubol

**Affiliations:** 1 Department of Veterinary Microbiology, Faculty of Veterinary Science, Chulalongkorn University, Henry Dunant Road, Pathumwan, Bangkok, 10330 Thailand; 2 Swine Viral Evolution and Vaccine Development Research Unit, Chulalongkorn University, Bangkok, Thailand; 3 Department of Pharmaceutics and Industrial Pharmacy, Faculty of Pharmaceutical Sciences, Chulalongkorn University, Bangkok, Thailand

**Keywords:** Vaccines, Microbiology

## Abstract

The objectives of the present study were to evaluate the immune response of six commercial vaccines against PRRSV-2 and PCV2, administered as monovalent or combined products via intramuscular (IM) or intradermal (ID) routes. Seventy-two, 3-week-old pigs were randomly allocated into 8 treatments with 9 pigs each: IMPP0/PCVMH7, IDPP0/PCVMH7, IMING0/PCVMH7, IMPP0/PCVMH0, IDPP0/PCVMH0, IMTRF0, NV/CH, and NV/NC. IMPP0/PCVMH0 and IMPP0/PCVMH7 groups were IM vaccinated once with Prime Pac PRRS (MSD Animal Health, The Netherlands) at 0 days post-vaccination (DPV), followed by single IM vaccination with Porcilis PCV M Hyo (MSD Animal Health, The Netherlands) either at 0 or 7 DPV, respectively. IDPP0/PCVMH0 and IDPP0/PCVMH7 groups were ID vaccinated once with Prime Pac PRRS (MSD Animal Health, The Netherlands) at 0 DPV, followed by a single concurrent ID injection of Porcilis PCV ID (MSD Animal Health, The Netherlands) and Porcilis M Hyo ID ONCE (MSD Animal Health, The Netherlands) either at 0 or 7 DPV, respectively. The IMING0/PCVMH7 group was IM vaccinated once with Ingelvac PRRS MLV (Boehringer Ingelheim, Germany) at 0 DPV, and subsequently IM vaccinated with Ingelvac CircoFLEX (Boehringer Ingelheim, Germany) and Ingelvac MycoFLEX (Boehringer Ingelheim, Germany) at 7 DPV. The IMTRF0 group was IM vaccinated once with combined products of Ingelvac PRRS MLV (Boehringer Ingelheim, Germany), Ingelvac CircoFLEX (Boehringer Ingelheim, Germany), and Ingelvac MycoFLEX (Boehringer Ingelheim, Germany) at 0 DPV. The NV/CH and NV/NC groups were left unvaccinated. At 28 DPV (0 days post-challenge, DPC), pigs were intranasally inoculated with a 4 ml of mixed cell culture inoculum containing HP-PRRSV-2 (10^5.6^ TCID_50_/ml) and PCV2d (10^5.0^ TCID_50_/ml). Antibody response, IFN-γ-secreting cells (SC), and IL-10 secretion in supernatants of stimulated PBMC were monitored. Sera were collected and quantified for the PRRSV RNA and PCV2 DNA using qPCR. Three pigs from each group were necropsied at 7 DPC, lung lesions were evaluated. Tissues were collected and performed immunohistochemistry (IHC). Our study demonstrated that concurrent vaccination via the ID or the IM route did not introduce additional reactogenicity. We found no interference with the induction of immune response between vaccination timing. In terms of an immune response, ID vaccination resulted in significantly lower IL-10 levels and higher IFN-γ-SC values compared to the IM-vaccinated groups. In terms of clinical outcomes, only one IM-vaccinated group showed significantly better efficacy when antigens were injected separately compared with concurrently. While the vaccines were ID delivered, these effects disappeared. Our findings confirm that concurrent vaccination of PRRSV-2 MLV and PCV2 via either the IM or the ID routes could be a viable immunization strategy to assist with the control of PRDC. In situations where maximal efficacy is required, over all other factors, concurrent vaccination is possible with the ID route but might not be an ideal strategy if using the IM route.

## Introduction

Amongst the pathogens listed by the World Organization for Animal Health^1^, the Porcine Reproductive and Respiratory Syndrome virus (PRRSV) and the Porcine Circovirus type 2 (PCV2) are the two most deleterious pathogens to the swine industry^2–4^. They were both recognized as significant pathogens during the 1990s in Europe and have established a global distribution since. The origin of the PRRSV has been well documented over the last decade in Europe^5^, and later named *Betaarterivirus suid 1*. The second species of PRRSV, named *Betaarterivirus suid 2*, has been identified in parallel in North America^6,7^, and then in Asia^8^, South America, and Europe^9^. On all continents, PRRSV strains of both species are present, with the recent remarkable emergence of a highly pathogenic variant of PRRSV-2 in China^10^. The case of PCV2 is comparable in the sense that it has attained global distribution, while its pleiotropic infectiousness has led to it being considered at the core of a range of clinical conditions: the PCV2-Associated Diseases (PCVADs)^11^. From a clinical point of view, PCV2 and PRRSV are frequently found coinfecting growing pigs, constituting the cornerstones of the Porcine Respiratory Disease Complex (PRDC)^12,13^.

Vaccines were rapidly developed against both these viruses and have been commercially available worldwide for the last 20 years. This has permitted a rapid and satisfactory control of PCVADs, even though the very high level of post-immunization protection in the global swine population may have resulted in a genetic shift of PCV2, possibly favoring subclinical infection^14^. A different situation is observed for PRRS, where viral elimination can be achieved at the farm level, but not on a wider geographic scale^15,16^. The three main reasons proposed to explain this are: the viral genome has a high mutation rate^17^, modified-live PRRS vaccines only provide limited heterologous protection^18,19^ while inactivated vaccines elicit limited protective immunity^20^ and airborne transmission is relevant in high-density farming regions^16,21^. Finally, co-infection of PCV2 and PRRSV has been proven to increase clinical severity (for a review, see^22^), and might even increase the mutation rate in PRRSV^23^.

Hence, efficacious control strategies are necessary at the farm level, and since veterinarians have imperfect tools at hand, an extensive exploration of the comparative efficacy of PRRSV modified-live vaccines (MLV) has been undertaken, showing that the development of neutralizing antibodies and virus shedding were vaccine-dependent^24^. Furthermore, in a challenge model, PRRSV-1 and PRRSV-2 MLVs were shown to decrease both viremia and lung lesions^25^. Finally, intradermal administration of vaccines induces, to a limited extent, cross-protection between the two PRRSV lineages. Vaccinations are hence a key tool used to control PCV2 and PRRSV. In Asia, they are also used to control many other endemic swine diseases, such as Classical Swine Fever and Foot and Mouth Disease. The complex disease situation results in pigs receiving multiple vaccinations throughout their life. Vaccination requires handlers to catch and restrain animals, increasing farm workload, and worker stress. Repeated handling of pigs has been demonstrated to be traumatic and has been associated with elevated cortisol levels^26^. Elevated cortisol in swine has been linked to decreased efficacy of both the innate and adaptive immune system^27^.

Many producers thus prefer to administer vaccination concurrently to reduce handling and hence stress. Concurrent vaccination is defined thus as the simultaneous, non-mixed injection of both PCV2 and PRRSV antigens in a vaccine. These vaccines are commonly administered to swine at weaning due to the epidemiological status of infection on numerous farms. Hence, a possible candidate for reduction of handling would be concurrently administering PRRSV and PCV2 vaccines. Vaccination against *Mycoplasma hyopneumoniae* is commonly carried out in the same period, as mentioned above in the field. Several commercial vaccines offer either ready-to-use or freshly mixed combinations with PCV2 and *Mycoplasma hyopneumoniae*, and some offering a three-way PCV2/*Mycoplasma hyopneumoniae*/PRRSV freshly mixed vaccine. Interestingly, despite the commonly combined vaccination timing, the course of field infection and the exposure to this pathogen exist during the fattening stage^28^, whereas the common period of PCV2 and PRRSV infections tends to occur at the nursery stage of production^29,30^.

Immune responses post-concurrent vaccination are usually vaccine-specific due to different combinations of antigen and adjuvant. Therefore, it is crucial to keep these factors constant when comparing vaccination routes and the timing of vaccination. Field studies evaluating such practices across different vaccines, vaccination routes, and timing are even more limited^31,32^. Some strains of wild-type PRRSV exhibit immunosuppressive properties and might harbor deleterious effects on other vaccines while administered concurrently^33^. The immunosuppressive effects of MLV-type PRRSV vaccines are a relatively unexplored field. In addition, simultaneous antigen saturation with multiple antigens showed a reduced immune response to vaccination in human children^34–36^. Nonetheless, this occurrence has not been demonstrated in swine vaccination.

In addition, the varying range of antigens, vaccination timing, and route of vaccination are also increasing. One alternative to traditional intramuscular vaccination methods is intradermal vaccination. Jet injectors are applied to produce a high-pressure stream of liquid^37^, forcing the vaccine into the dermis of the pig without the use of needles. There is an increase in commercially available veterinary medical devices that can deliver vaccines intradermally into the skin. Intradermal vaccination in swine targets antigen-presenting cells (APCs) in the epidermis of the pig, potentially inducing cell-mediated immunity better than that of muscle tissue^38^. This has been associated with equivalent or with better immune responses post vaccination^39–41^. Intradermal vaccination of swine has also demonstrated other benefits through needle removals, such as reduced needle-stick injuries to staff^42^ and reduced iatrogenic transmission of viruses^43^.

In summary, there is a need to understand how a multitude of variables (vaccination timing, number of antigens, and vaccination route) interact to produce clinical outcomes. Producers need to balance out the reduction of stress and decreased workload associated with concurrent vaccination and vaccination events with the overall immune response post-vaccination with multiple antigens. Therefore, the rationale for this study is to evaluate the immune response of experimental pigs in a model mimicking field conditions with different vaccination timing and routes against PRRSV-2 and PCV2, followed by evaluating the protection level of these strategies with a simultaneous PRRSV-2/PCV2 challenge.

## Materials and methods

### Experimental design

All animal procedures in the present study were conducted in accordance with the recommendation in the Guild for the Care and Use of Laboratory Animal of the National Research Council of Thailand according to protocols reviewed and approved by the Chulalongkorn University Animal Care and Use Committee (protocol number 2031015). All methods were performed in accordance with the relevant guidelines and regulations. The study is reported in accordance with the ARRIVE guidelines (https://arriveguidelines.org).

Seventy-two, castrated male, PRRSV-free with 3-week-old pigs were purchased from PRRSV-free commercial herds. Upon arrival, pigs were acclimated for 3 days and tested for the presence of PRRSV and other pathogens with real-time PCR and ELISA kits. Pigs were randomly allocated into 8 treatment groups with 9 pigs each: IMPP0/PCVMH7, IDPP0/PCVMH7, IMING0/PCVMH7, IMPP0/PCVMH0, IDPP0/PCVMH0, IMTRF0, NV/CH, and NV/NC (Table 1).

**Table 1:** Experimental design in the present study.

Treatment group	Number of pigs	Route of vaccination	PRRSV MLV vaccination	PCV2/M. Hyo vaccination	PRRSV MLV and dose	PCV2-based vaccines and dose	M. Hyo-based vaccine and dose
IMPP0/PCVMH7	9	IM	0 DPV	7 DPV	Prime Pac PRRS, 1 ml	Porcilis PCV M Hyo, 2 ml	–
IDPP0/PCVMH7	9	ID	0 DPV	7 DPV	Prime Pac PRRS, 0.2 ml	Porcilis PCV ID, 0.2 ml	Porcilis M Hyo ID ONCE, 0.2 ml
IMING0/PCVMH7	9	IM	0 DPV	7 DPV	Ingelvac PRRS MLV, 2 ml	Ingelvac CircoFLEX, 1 ml	Ingelvac MycoFLEX, 1 ml
IMPP0/PCVMH0	9	IM	0 DPV	0 DPV	Prime Pac PRRS, 1 ml	Porcilis PCV M Hyo, 2 ml	–
IDPP0/PCVMH0	9	ID	0 DPV	0 DPV	Prime Pac PRRS, 0.2 ml	Porcilis PCV ID, 0.2 ml	Porcilis M Hyo ID ONCE, 0.2 ml
IMTRF0	9	IM	0 DPV	0 DPV	Ingelvac 3FLEX, 2 ml	–	–
NV/CH	9	–	–	–	–	–	–
NV/NC	9	–	–	–	–	–	–

Eight treatment groups include 6 vaccinated groups and 2 non-vaccinated groups. Routes of vaccine administration were included either intramuscular (IM) or intradermal (ID) using IDAL 3G vaccinator.

*DPV* days post-vaccination.

Pigs in the IMPP0/PCVMH0 and IMPP0/PCVMH7 groups were intramuscularly (IM) vaccinated once with a 1 ml dose of Prime Pac PRRS (PRRSV-2 MLV, MSD Animal Health, Boxmeer, The Netherlands) at 0 days post-vaccination (DPV), followed by a single IM vaccination once with a 2 ml dose of Porcilis PCV M Hyo (MSD Animal Health, Boxmeer, The Netherlands), either at 0 or at 7 DPV, respectively. In contrast, pigs in the IDPP0/PCVMH0 and IDPP0/PCVMH7 groups were intradermally (ID) vaccinated once with a 0.2 ml dose of Prime Pac PRRS (PRRSV-2 MLV, MSD Animal Health, Boxmeer, The Netherlands) at 0 DPV, followed by concurrent ID vaccination once with a 0.2 ml dose of Porcilis PCV ID (MSD Animal Health, Boxmeer, The Netherlands) and a 0.2 ml dose of Porcilis M Hyo ID ONCE (MSD Animal Health, Boxmeer, The Netherlands), either at 0 or at 7 DPV, respectively. ID vaccination was performed using IDAL 3G needle-free device (MSD Animal Health, Boxmeer, The Netherlands).

Pigs in the IMING0/PCVMH7 group were IM vaccinated once with a 2 ml dose of Ingelvac PRRS MLV (PRRSV-2 MLV, Boehringer Ingelheim, Rhien, Germany) at 0 DPV. Subsequently, pigs in this group were IM vaccinated once with a combined dose of 1 ml Ingelvac CircoFLEX (Boehringer Ingelheim, Rhien, Germany) and 1 ml of Ingelvac MycoFLEX (Boehringer Ingelheim, Rhien, Germany) at 7 DPV. Pigs in the IMTRF0 group were IM vaccinated once with a 2 ml dose of combined products of Ingelvac PRRS MLV (PRRSV-2 MLV, Boehringer Ingelheim, Rhien, Germany), Ingelvac CircoFLEX (Boehringer Ingelheim, Rhien, Germany), and Ingelvac MycoFLEX (Boehringer Ingelheim, Rhien, Germany) at 0 DPV. Pigs in the NV/CH and NV/NC groups were left unvaccinated.

At 28 DPV (0 days post-challenge, DPC), pigs were intranasally inoculated with a 4 ml of mixed cell culture inoculum containing HP-PRRSV-2 (FDT10US23 isolate, fifth passage of MARC-145 cells at 10^5.6^ TCID_50_/ml) and PCV2 (S1918STC isolate, third passage of PK-15 cells at 10^5.0^ TCID_50_/ml), with 2 ml/nostril. Pigs in the NV/NC group were left as a non-vaccination, non-challenge control group. All pigs were housed in separated rooms with separated airflow spaces. Clinical signs and rectal temperatures were daily recorded throughout the experiment.

Blood samples were collected at 0, 7, 14, 21 and 28 DPV, 3, 5, 7, 10, 14, 21 and 28 DPC. Sera were separated and assayed for the presence of PRRSV- and PCV2-specific antibodies using ELISA kits. Genomic copy numbers of PRRSV and PCV2 were quantified using RT-qPCR. Peripheral blood mononuclear cells (PBMC) were isolated and used for in vitro stimulation to measure IL-10 secretion using an ELISA kit and virus-specific IFN-γ-secreting cells (IFN-γ-SC) using an ELISPOT assay. At 7 DPC, three pigs from each group were euthanized and necropsied. Pneumonic lung lesions were evaluated as previously described^44^. Tissues were collected, PRRSV- and PCV2-specific antigens were evaluated using immunohistochemistry (IHC).

### Vaccines, vaccination, and viruses

Vaccines used for vaccination were two of each PRRSV, PCV2, and *Mycoplasma (M.) hyopneumoniae* vaccines. PRRSV vaccines used for vaccination were two PRRSV-2 MLVs including Prime Pac PRRS (MSD Animal Health, Boxmeer, The Netherlands) and Ingelvac PRRS MLV (Boehringer Ingelheim, Rhien, Germany). Prime Pac PRRS is available in two different preparations for intramuscular (IM) or intradermal (ID) vaccination. For PCV2 and *M. hyo* vaccination, vaccines were including Porcilis PCV ID (MSD Animal Health, Boxmeer, The Netherlands) and Ingelvac CircoFLEX (Boehringer Ingelheim, Rhien, Germany), Porcilis M Hyo ID ONCE (MSD Animal Health, Boxmeer, The Netherlands), Porcilis PCV M Hyo (MSD Animal Health, Boxmeer, The Netherlands) and Ingelvac MycoFLEX (Boehringer Ingelheim, Rhien, Germany), respectively. Dosage and administration routes were applied following the manufacturer’s instructions.

Briefly, a 2 ml dose of Ingelvac PRRS MLV (batch number 2451281A) and a 1 ml dose of Prime Pac PRRS (batch number A605CE04) was used for IM vaccination, respectively. A 0.2 ml dose of Prime Pac PRRS MLV (batch number A605CE04) was used for ID vaccination using IDAL 3G needle-free device. A 1 ml dose of Ingelvac CircoFLEX (batch number 3091225A), a 1 ml dose of Ingelvac MycoFLEX (batch number 2730552A), and a 2 ml dose of Porcilis PCV M Hyo (batch number A099A01) were used for IM vaccination. A 0.2 ml dose of Porcilis PCV ID vaccine (batch number A020A01), and Porcilis M Hyo ID ONCE vaccine (batch number A027B01) were used for ID vaccination. ID vaccination was performed using IDAL 3G needle-free device (MSD Animal Health, Boxmeer, The Netherlands).

The combined vaccine was prepared as follow: Ingelvac MycoFLEX (batch number 2730552A) and Ingelvac CircoFLEX (batch number 3091225A) were mixed, and the mixture was then used to rehydrate Ingelvac PRRS MLV (batch number 2451281A). This was done in place of the Ingelvac PRRS MLV accompanying vaccine diluent and against the manufacturer’s mixing condition. A 2 ml dose of combined vaccine was used for IM vaccination.

Homologous vaccine viruses, highly pathogenic (HP)-PRRSV-2 and PCV2 isolates were used in the present study. Homologous vaccine viruses refer to vaccine strains that were used as recall antigens for in vitro stimulation assay in the measurement of virus-specific IFN-γ-SC and IL-10 secretion were performed in previously described methods^24,25^. To challenge pigs, a mixed cell culture supernatant containing Thai HP-PRRSV-2 isolate FDT10US23 (fifth passage in MARC-145 cells) and PCV2 isolate S1918STC (third passage in PK-15 cells) was used as a virus inoculum. The isolate FDT10US23 is an HP-PRRSV-2 variant genetically classified in the sublineage 8.7/HP-PRRSV-2 based on international systematic classification, according to the previously described method^45^. The ORF5 genome sequence is available in GenBank under accession number JN255836. The isolates FDT10US23 and S1918STC were isolated from swine herds experiencing PRRS outbreaks in the western region of Thailand during 2010–2011^46,47^. Pathogenesis and challenge studies of the challenged isolate were demonstrated according to previous studies^25,30,46,48,49^.

### Clinical signs and rectal temperature recording

Clinical sings and rectal temperature were monitored daily post-vaccination (DPV) and post-challenge periods (DPC) following two consecutive weeks by the same personnel at the same time. The severity of respiratory disease was evaluated using a scoring system for each pig following stress induction as previously described^44^: 0 = normal, 1 = mild dyspnea and/or tachypnea when stressed, 2 = mild dyspnea and/or tachypnea when at rest, 3 = moderate dyspnea and/or tachypnea when stressed, 4 = moderate dyspnea and/or tachypnea when at rest, 5 = severe dyspnea and/or tachypnea when stressed, and 6 = severe dyspnea and/or tachypnea when at rest.

### Antibody detection

PRRSV- and PCV2-specific antibodies were measured using commercially available ELISA kits: IDEXX PRRS X3 Ab test (IDEXX Laboratories Inc., MA, USA) and BioCheck PCV2 ELISA (BioCheck BV, Reeuwijk, The Netherlands), and serum neutralization (SN) assay. The ELISA assays were performed following the manufacturer’s recommendations. Sera were considered positive for PRRSV if the S/P ratio was greater than 0.4 and positive for PCV2 if the titer was greater than 1070, respectively.

### Isolation of porcine peripheral blood mononuclear cells (PBMC)

Peripheral blood mononuclear cells (PBMC) were isolated from heparinized blood using gradient density centrifugation (Lymphosep™, Biowest, MO, USA) as previously described^39^. Isolated PBMC were counted by an inverted microscope, and cell concentrations were accessed in cRPMI-1640 medium [RPMI-1640 supplemented with 10% fetal bovine serum (FBS), 2 mM l-glutamine, and 50 μg/ml of gentamycin]. The viability of isolated PBMC was determined by Trypan blue (Sigma-Aldrich, MO, USA) staining and more than 90% viability was used for in vitro stimulation for IL-10 production and enzyme-linked immunospot (ELISPOT) assay as described below.

### Porcine interleukin-10 (IL-10)

Porcine IL-10 concentration in the supernatant of stimulated PBMC was quantified using a porcine ELISA IL-10 kit (R&D System, MN, USA) under the manufacturer’s instructions and previously described methods^43,48^. In brief, total 2 × 10^6^ PBMC were seed into 96-well plates and cultured in vitro for 24 h with either homologous vaccine viruses at 0.01 multiplicity of infection (MOI), phytohemagglutinin (PHA, 10 μg/ml, Sigma-Aldrich, MO, USA), or MARC-145 cell lysate (mock suspension). In each pig, the levels of porcine IL-10 secretion were calculated by subtracting the value of mock-stimulated well from the PRRSV-stimulated well. Subtracted values were compared between treatment groups.

### ELISPOT assay

The number of virus-specific IFN-γ-SC were determined in stimulated PBMC using a commercial ELISPOT IFN-γ kit (ELISpot porcine IFN-γ, R&D System, MN, USA). The assay was performed in accordance with manufacturer’s instructions and a previously described method^43,48^. Briefly, 2 × 10^5^ PMBC/well were seeded into 96-well plates and stimulated with either homologous vaccine viruses or heterologous viruses (FDT10US23 and S1918STC isolates) at 0.01 MOI for 24 h. Phytohemagglutinin (PHA) and cRPMI-1640 were used as positive and negative controls, respectively. The spots were counted by an automated ELISPOT Reader (AID ELISPOT Reader, AID GmbH, Strassberg, Germany). The background values were subtracted from the respective count of the stimulated cells and the immune response was expressed as the number of virus-specific IFN-γ-SC per 10^6^ PBMC.

### Quantification of PRRSV RNA

Total RNA was extracted from sera using NucleoSpin RNA Virus (Macherey–Nagel, Duren, Germany) according to the manufacturer’s instructions. The RNA quality was measured using a NanoDrop spectrophotometer (Colibri spectrophotometer, Titertek Berthold, Pforzheim, Germany). Copy number of PRRSV RNA in sera and nasal swabs was quantified using probed-based real-time PCR as previously described^48^. The reaction was carried out in QuantStudio 3 Real-time PCR machine (Thermo-Fisher Scientific, MA, USA).

### Quantification of PCV2 DNA

Total DNA was extracted from sera using NucleoSpin Tissue (Macherry–Nagel, Duren, Germany) according to the manufacturer’s instructions. The DNA quality was measured using a NanoDrop spectrophotometer (Colibri spectrophotometer, Titertek Berthold, Pforzheim, Germany) and quantified using QuantStudio 3 Real-time PCR machine (Thermo-Fisher Scientific, MA, USA). The capsid gene of PCV2-specifc primers were used as follow: forward primer 5′-GTGCCCGCTGCCACATCGAG-3′; reverse primer 5′-CAAAAGTTCAGCCAGCCCGCGGA-3′. The reaction contained 0.1 μg of DNA, 0.2 μM of each primer, 2′ qPCRBIO SyGreen Mix (PCR Biosystems, London, UK), and deionized water to yield a 20 μl final volume. The thermal profile was 94 °C for 4 min, followed by 35 cycles of 94 °C for 45 s, 60 °C for 30 s, and fluorescence acquisition at 72 °C for 45 s. pGEM-T Easy Vector (Promega, WI, USA) containing an inserted capsid gene was used to construct plasmid standard. A standard curve was generated using serial diluted plasmid standards of 10^0^–10^7^ copies/μl. Copy number of PCV2 DNA was calculated using standard curve method.

### Pathological examination

Pigs from each group were necropsied at 7 DPC. Macroscopic and microscopic lung lesions associated with PRRSV-induced pneumonia were evaluated as previously described^44^. For macroscopic lung lesions, each lung lobe was assigned a number to represent the approximate percentage of the volume of the entire lung and the percentage of the volume from each lobe added to the entire lung score (range from 0 to 100% of affected lung). Additionally, the size of lymph nodes was scored ranging from 0 (normal) to 3 (four times the normal size) according to previously described method^50^. Sections were collected from all lung lobes and lymph nodes as previously described^44,50^ and fixed with 10% neutral buffered formalin for 7 days. Tissues were routinely processed and embedded in paraffin in an automated tissue processor. Sections were cut and stained with hematoxylin and eosin (H&E). For microscopic lung lesions, the lung sections were examined in a blinded manner and given an estimated score of the severity of interstitial pneumonia. In brief, 0 = normal; 1 = mild interstitial pneumonia; 2 = moderate multifocal interstitial pneumonia; 3 = moderate diffuse interstitial pneumonia, and 4 = severe diffuse interstitial pneumonia. The mean values of the microscopic lung lesion score of each group were calculated.

### Immunohistochemistry (IHC)

Immunohistochemistry for PRRSV antigens in lung sections were performed using PRRSV-specific monoclonal antibody as previously described^25^. In brief, tissues were processed and placed on Superfrost Plus slides (Thermo Fisher Scientific, MA, USA). Sections were deparaffinized, rehydrated, and treated with proteinase K (Thermo Fisher Scientific, MA, USA). Endogenous alkaline phosphatase was quenched with 0.3% hydrogen peroxide. Slides were treated with BSA and incubated with monoclonal antibody overnight. After washing, PRRSV antigens were visualized by secondary antibody HRP-conjugated (Agilent, Santa Clara, CA, USA) and counterstained with Meyer’s hematoxylin. To obtain quantitative data, slides were analyzed with the NIH Image J 1.50i Program (https://rsb.info.nih.gov/ij). In each slide, 10 fields were randomly selected, and the number of positive cells per unit area (0.95 mm^2^) was determined as previously described^25,51,52^. The mean values were calculated.

PCV2 antigens were analyzed by IHC using paraffin-embedded blocks of tissues including lungs, tonsils, tracheobronchial, and inguinal lymph nodes. In brief, tissues were processed and placed on Superfrost Plus slides (Thermo Fisher Scientific, MA, USA). Sections were deparaffinized, rehydrated, and treated with proteinase K (Thermo Fisher Scientific, MA, USA). Endogenous alkaline phosphatase was quenched with 0.3% hydrogen peroxide. Slides were treated with BSA and incubated with PCV-2 specific monoclonal antibody (PCV-2-A, RIT, SD, USA) overnight. After washing, PCV2 antigens were visualized by secondary antibody HRP-conjugated (Agilent, Santa Clara, CA, USA) and counterstained with Meyer’s hematoxylin.

The scoring of PCV2 antigen was performed according to previously described^50^. Assessment of staining for PCV2 antigen was done in a blinded fashion and scores ranged from 0 to 3 (0 = negative; 1 = less than 10% of the lymphoid follicles have cells with PCV2 antigen staining; 2 = 10–50% of the lymphoid follicles contain cells with PCV2 antigen staining; 3 = more than 50% of the lymphoid follicles contain cells with PCV2 antigen. Mean values were calculated.

### Statistical analysis

Analysis of variance (ANOVA) was performed to determine if there were significant differences among groups for each day separately. If the *p*-value for an ANOVA table was less than or equal to 0.05, the difference between treatment groups was evaluated using a multiple comparison test. All data were analyzed using IBM SPSS Statistic for Windows version 22.0 (IBM, Armonk, NY, USA) (https://www.ibm.com/software/analytics/spss/register/).

## Results

### Rectal temperature and clinical sings

**Figure 1:**
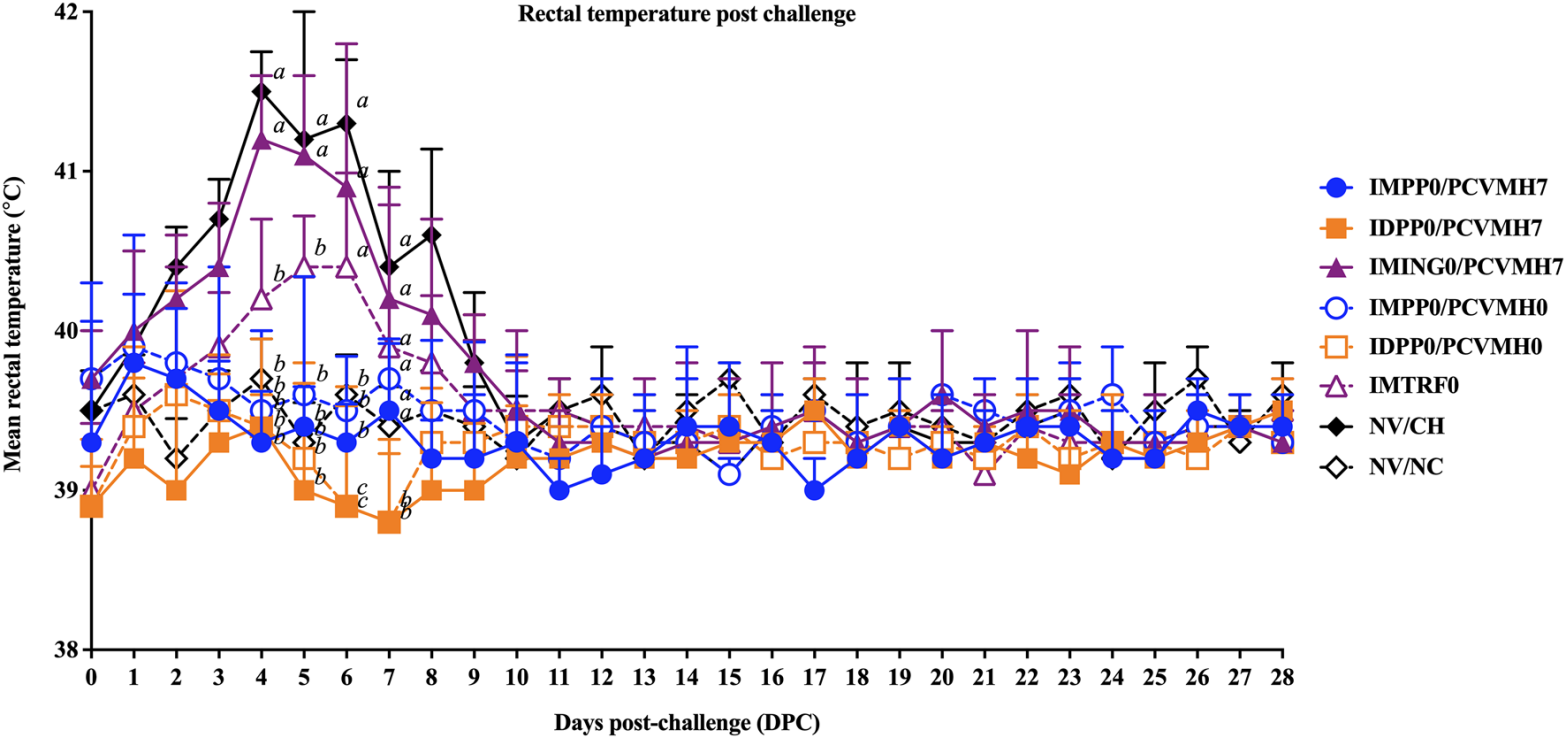
Mean values of rectal temperature post-challenge. Values expressed as mean ± SEM. The results were compared using two-way ANOVA for multiple comparisons. Different lowercase letters (a–c) indicate significant differences between treatment groups (*p* < 0.05) for each time-point.

No clinical signs were observed in the NV/NC group throughout the experiment. Following vaccination, the rectal temperature of pigs in all vaccinated groups were in a physiological range and showed no clinical signs. Following challenge, significant differences in the rectal temperature among groups were observed from 4 to 7 DPC, and ID-vaccinated groups had significantly (*p* < 0.05) lower rectal temperatures compared to that of the IM-vaccinated groups from 6 to 7 DPC. The IMING0/PCVMH7 and NV/CH groups showed mild- to severe respiratory disease and had significantly (*p* < 0.05) higher rectal temperatures than that of the other groups at 4 and 5 DPC (Fig. 1). At 6 and 7 DPC, pigs in the IDPP0/PCVMH7 and IDPP0/PCVMH0 groups had significantly (*p* < 0.05) lower rectal temperatures compared to not only the IM-vaccinated groups, but also compared with that of the NV/NC group. There was no difference in the rectal temperature among groups from 8 to 28 DPC.

### PRRSV-specific antibody response

**Figure 2:**
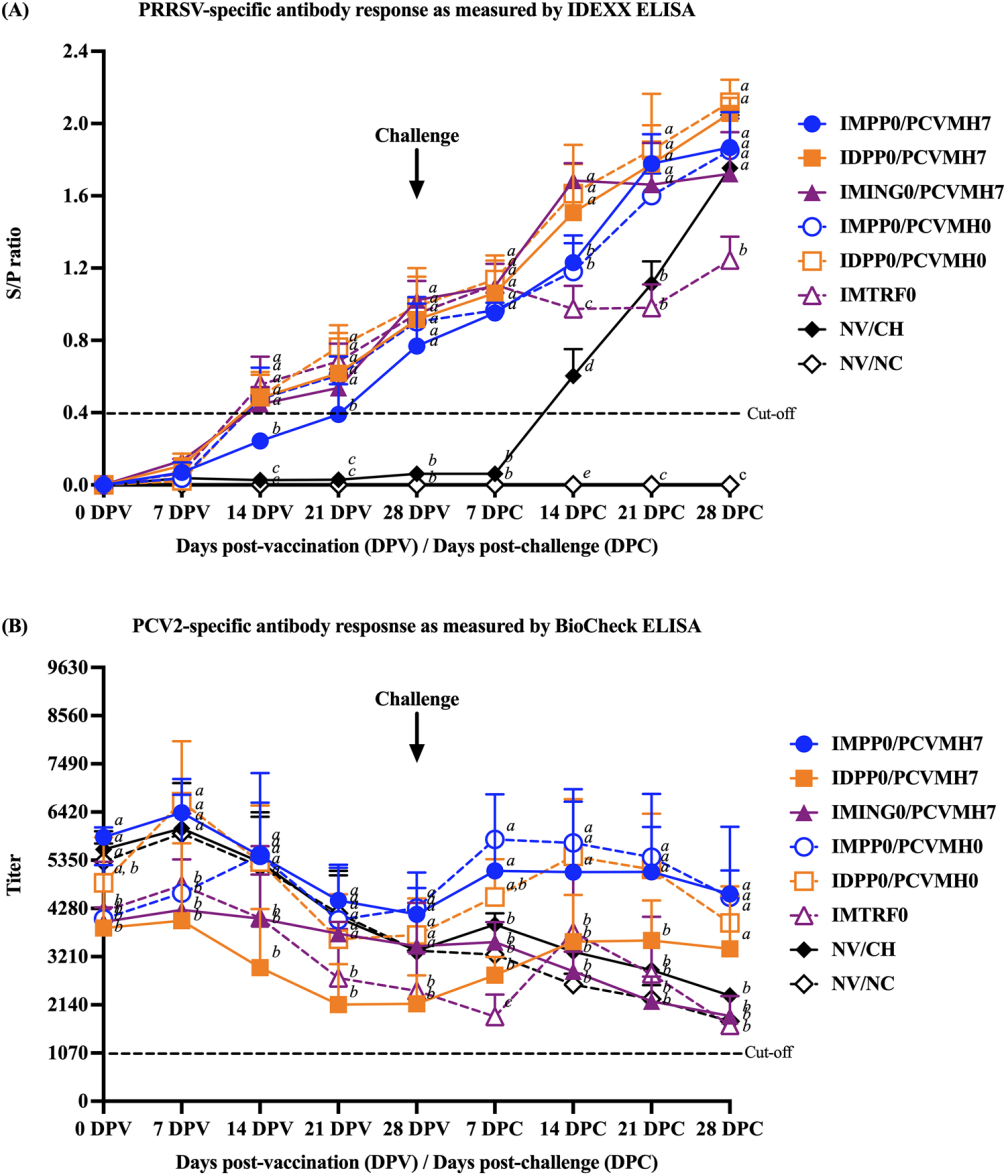
Mean values of (**A**) PRRSV-specific antibody response and (**B**) PCV2-specific antibody response as measured by ELISA. Values expressed as mean ± SEM. (**A**) Sample-to-positive (S/P) ratios equal to or greater than 0.4 were considered positive (cut-off value, dashed-line). (**B**) PCV2-specific titers equal to or greater than 1070 were considered positive (cut-off value, dashed-line). The results were compared using two-way ANOVA for multiple comparisons. Different lowercase letters (a–c) indicate significant differences between treatment groups (*p* < 0.05) for each time-point.

PRRSV-specific antibody response as measured by ELISA was shown in Fig. 2A. Regardless of vaccine use, route, and timing of administration, all vaccinated groups had slightly increased the PRRSV-specific antibody response at 7 DPV but remained negative for PRRS (S/P ratio < 0.4). Antibody titers continually increased at 14 DPV and reached high levels until 28 DPC. Following vaccination, there was no difference in the antibody response among vaccinated groups from 7 to 28 DPV. Except for the IMPP0/PCVMH7 group, the antibody response was significantly (*p* < 0.05) lower than that of other groups at 14 and 21 DPV. Following challenge, the PRRSV-specific antibody response was not different among vaccination groups at 7 DPC. At 14 DPC, the IDPP0/PCVMH7, IMING0/PCVMH7, and IDPP0/PCVMH0 groups had significantly (*p* < 0.05) higher antibody titers than that of the other vaccinated groups. Meanwhile, the IMTRF0 group had a significantly (*p* < 0.05) lower antibody titer than that of the other vaccinated groups at 14 DPC. There was no difference in the antibody response among vaccinated groups at 21 and 28 DPC. Except for the IMTRF0 group, the antibody response was significantly (*p* < 0.05) lower than that of the other vaccinated groups at 21 and 28 DPC (Fig. 2A). No antibody titers were detected in pigs of the NV/NC group throughout the experiment.

### PCV2-specific antibody response

PCV2-specific antibody response as measured by ELISA was shown in Fig. 2B. PCV2-specific antibodies were detected in all vaccinated groups at 0 DPV in which the IMPP0/PCVMH7, NV/CH, and NV/NC groups had significantly (*p* < 0.05) higher titers than that the IDPP0/PCVMH7, IMING0/PCVMH7, IMPP0/PCVMH0, and IMTRF0 groups. The PCV2-specific antibody titers of the NV/CH and NV/NC groups continually decreased to basal levels over time until 28 DPC. Following vaccination, similar patterns of PCV2 antibody response were detected among vaccinated groups which continually decreased from 7 DPV and dropped to the basal levels at 28 DPV regardless of the vaccine use, route, and timing of administration. Except for one ID-vaccinated group, the slightly increased PCV2 antibody was detected in the IDPP0/PCVMH0 group at 7 DPV. The PCV2-specific antibody titers of the IDPP0/PCVMH7, IMING0/PCVMH7, IMPP0/PCVMH0, and IMTRF0 groups were significantly (*p* < 0.05) lower than that of the other groups at 7 DPV. At 14 DPV, the IDPP0/PCVMH7, IMING0/PCVMH7, and IMTRF0 groups had significantly (*p* < 0.05) lower PCV2-specific antibody titers than that the other groups. Whereas the IDPP0/PCVMH7 and IMTRF0 groups had significantly (*p* < 0.05) lower PCV2 antibody titers than that of the other groups at 21 and 28 DPV.

Following challenge, increased PCV2-specific antibody titers were notably observed in all vaccinated groups. Except for the IMING0/PCVMH7 group that showed decrease in the PCV2 antibody titer until 28 DPC. At 7 DPC, the IMTRF0 group had a significantly (*p* < 0.05) lower PCV2 antibody titer than that of the other groups. Pigs in the IMPP0/PCVMH7, IMPP0/PCVMH0, and IDPP0/PCVMH0 groups had significantly (*p* < 0.05) higher PCV2 antibody titers than that of the other groups from 14 to 21 DPC. While the PCV2 antibody titers of IMING0/PCVMH7 and IMTRF0 groups had significantly (*p* < 0.05) lower levels compared to that of the other groups at 28 DPC (Fig. 2B).

### Porcine IL-10 production

**Figure 3:**
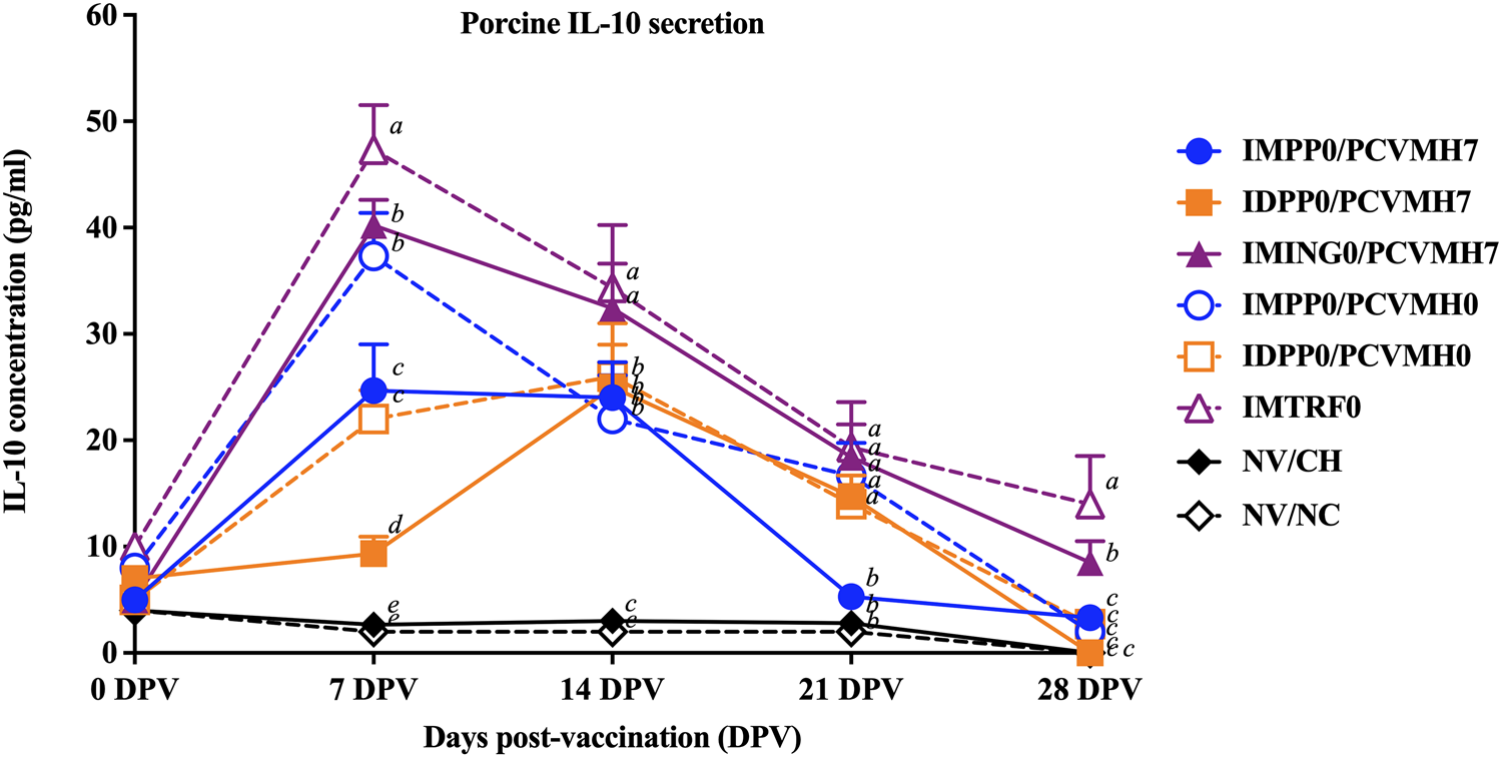
Quantification of porcine IL-10 secretion in stimulated PBMC with homologous viruses (vaccine viruses). Values expressed as mean ± SEM. The results were compared using two-way ANOVA for multiple comparisons. Different lowercase letters (a–d) indicate significant differences between treatment groups (*p* < 0.05) for each time-point.

Increased IL-10 levels were first detected in all vaccinated groups at 7 DPV and continually decreased to basal levels until 28 DPV (Fig. 3). The induction of IL-10 secretion from the cell culture supernatants of stimulated PBMC was markedly detected in the IM-vaccinated groups that reached the highest values at 7 DPV then gradually declined until 28 DPV. Meanwhile, IL-10 secretion in the ID-vaccinated groups continually increased and reached peaks at 14 DPV. The IL-10 levels then continually decreased to the basal levels at 28 DPV. Notably, concurrent vaccination within the same day (day 0/0) showed relatively higher IL-10 secretion than that of the vaccination at day 0/7.

At 7 DPV, pigs in the IMTRF0 group had the highest (*p* < 0.05) IL-10 levels (47.3 ± 4.19 pg/ml) compared to those of the other groups. Meanwhile, the IDPP0/PCVMH7 group had the significantly lowest IL-10 level (9.3 ± 1.6 pg/ml) than that of the other vaccinated groups at 7 DPV. Pigs in the IMING0/PCVMH7 and IMPP0/PCVMH0 groups had significantly higher IL-10 levels (40.2 ± 2.4 and 37.3 ± 4.0 pg/ml) compared to that of the IMPP0/PCVMH7 (24.6 ± 4.3 pg/ml) and IDPP0/PCVMH0 (22.0 ± 2.7 pg/ml) groups at 7 DPV. At 14 DPV, the IMING0/PCVMH7 and IMTRF0 groups had significantly (*p* < 0.05) higher IL-10 levels (32.4 ± 4.2 and 34.3 ± 5.9 pg/ml) than that of the other groups. At 21 DPV, the IMPP0/PCVMH7 group was the lowest in IL-10 levels (5.3 ± 1.0 pg/ml) than that of the other groups. While the IL-10 secretion in the IMPP0/PCVMH7, IMPP0/PCVMH0, IDPP0/PCVMH7 and IDPP0/PCVMH0 groups were significantly (*p* < 0.05) lower levels than that of the other groups at 28 DPV (Fig. 3).

### PRRSV-specific IFN-γ-SC

**Figure 4:**
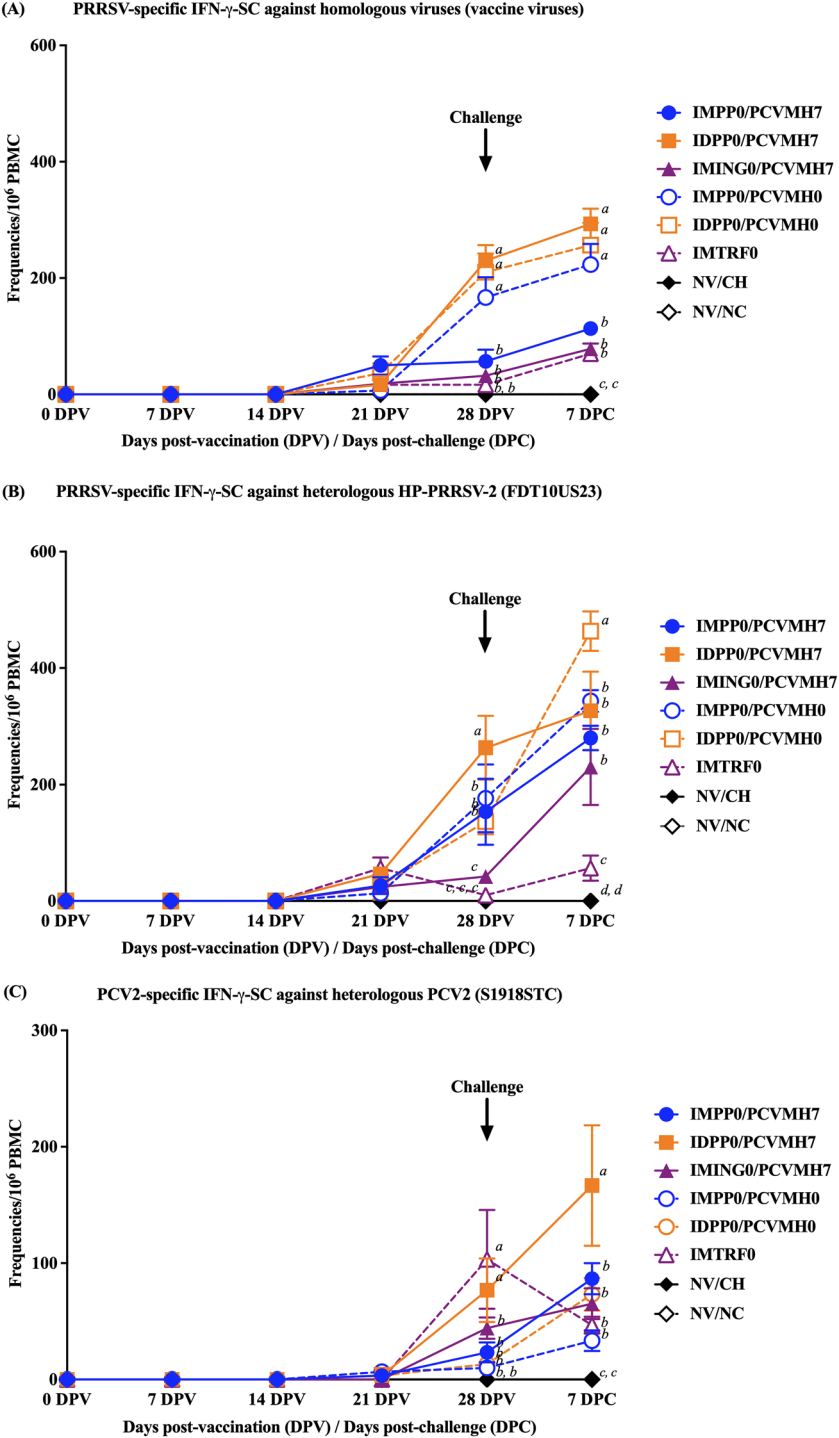
Frequencies of virus-specific IFN-γ-SC after stimulation with (**A**) homologous viruses (vaccine viruses), (**B**) heterologous HP-PRRSV-2 (FDT10US23) virus, and (**C**) heterologous PCV2 (S1918STC) virus. Values expressed as mean ± SEM. The results were compared using two-way ANOVA for multiple comparisons. Different lowercase letters (a–d) indicate significant differences between treatment groups (*p* < 0.05) for each time-point.

No PRRSV-specific IFN-γ-SC detected in the NV/CH and NV/NC groups throughout the experiment. The induction of PRRSV-specific IFN-γ-SC was remarkedly observed in the ID-vaccinated groups regardless of vaccine use and timing of administration that showed a significantly higher frequency compared to that of the IM-vaccinated group (Fig. 4). For homologous vaccine virus stimulation (Fig. 4A), the PRRSV-specific IFN-γ-SC were first detected at 21 DPV in all vaccinated groups, but the levels were low (ranged from 16.7 ± 14.5 to 36.7 ± 16.7 cells/10^6^ PBMC). Then, the frequencies of PRRSV-specific IFN-γ-SC were gradually increased from 21 DPV until 7 DPC in which statistical differences were observed at 28 DPV and 7 DPC. The IDPP0/PCVMH7, IMPP0/PCVMH0 and IDPP0/PCVMH0 groups had significantly (*p* < 0.05) higher frequencies of IFN-γ-SC (230 ± 26.5, 166.7 ± 34.8 and 210 ± 32.1 cells/10^6^ PBMC) than those in the IMPP0/PCVMH7, IMING0/PCVMH7 and IMTRF0 groups (56.7 ± 20.0, 32.2 ± 4.2 and 16.7 ± 8.8 cells/ 10^6^ PBMC) at 28 DPV. While the IMPP0/PCVMH7, IMING0/PCVMH7 and IMTRF0 groups (113.3 ± 8.8, 78.3 ± 12.1 and 70.0 ± 17.3 cells/10^6^ PBMC) had significantly (*p* < 0.05) lower frequencies of PRRSV-specific IFN-γ-SC than those in the IDPP0/PCVMH7, IMPP0/PCVMH0 and IDPP0/PCVMH0 groups (293.3 ± 26.0, 223.3 ± 35.3 and 256.7 ± 38.4 cells/10^6^ PBMC) at 7 DPC (Fig. 4A).

For heterologous HP-PRRSV-2 (FDT10US23) stimulation, the PRRSV-specific IFN-γ-SC was first detected at 21 DPV in all vaccinated groups (ranged from 24.2 ± 7.2 to 56.7 ± 18.6 cells/10^6^ PBMC) without statistical differences (Fig. 4B). Like homologous virus stimulations, the PRRSV-specific IFN-γ-SC against heterologous HP-PRRSV-2 were then gradually increased from 21 DPV until 7 DPC which greatly observed in ID-vaccinated groups. At 28 DPV, the IDPP0/PCVMH7 group had a significantly (*p* < 0.05) higher frequencies of IFN-γ-SC (263.3 ± 54.6 cells/10^6^ PBMC) than those in the other groups. While the IMING0/PCVMH7 and IMTRF0 groups (42.1 ± 11.2 and 10.0 ± 5.8 cells/10^6^ PBMC) had significantly (*p* < 0.05) lower frequencies of IFN-γ-SC than that of the other groups at 28 DPV. At 7 DPC, the IDPP0/PCVMH0 group (463.3 ± 33.9 cells/10^6^ PBMC) had a significantly higher frequency of PRRSV-specific IFN-γ-SC than that of the other vaccinated groups. In contrast, the IMTRF0 group had a significantly (*p* < 0.05) lower frequency of PRRSV-specific IFN-γ-SC (56.8 ± 21.9 cells/10^6^ PBMC) than that of the other groups at 7 DPC (Fig. 4B).

### PCV2-specific IFN-γ-SC

No PCV2-specific IFN-γ-SC against heterologous PCV2 recall antigen detected in the NV/CH and NV/NC groups throughout the experiment. The IFN-γ-SC against heterologous PCV2 recall antigen (S1918STC) were relatively low levels compared with the PRRSV recall antigens (Fig. 4C). PCV2-specific IFN-γ-SC were first detected at 28 DPV and reached the peaks at 7 DPC. Pigs in the IDPP0/PCVMH0 and IMTRF0 groups had significantly (*p* < 0.05) higher frequencies of PCV2-specific IFN-γ-SC (76.67 ± 27.2 and 103.3 ± 42.5 cells/10^6^ PBMC) than that of the other groups at 28 DPV. In contrast, the IDPP0/PCVMH7 group had a significantly (*p* < 0.05) higher frequencies of PCV2-specific IFN-γ-SC (166.7 ± 51.7 cells/10^6^ PBMC) than that of the other groups at 7 DPC.

### Quantification of PRRSV RNA in blood

**Figure 5:**
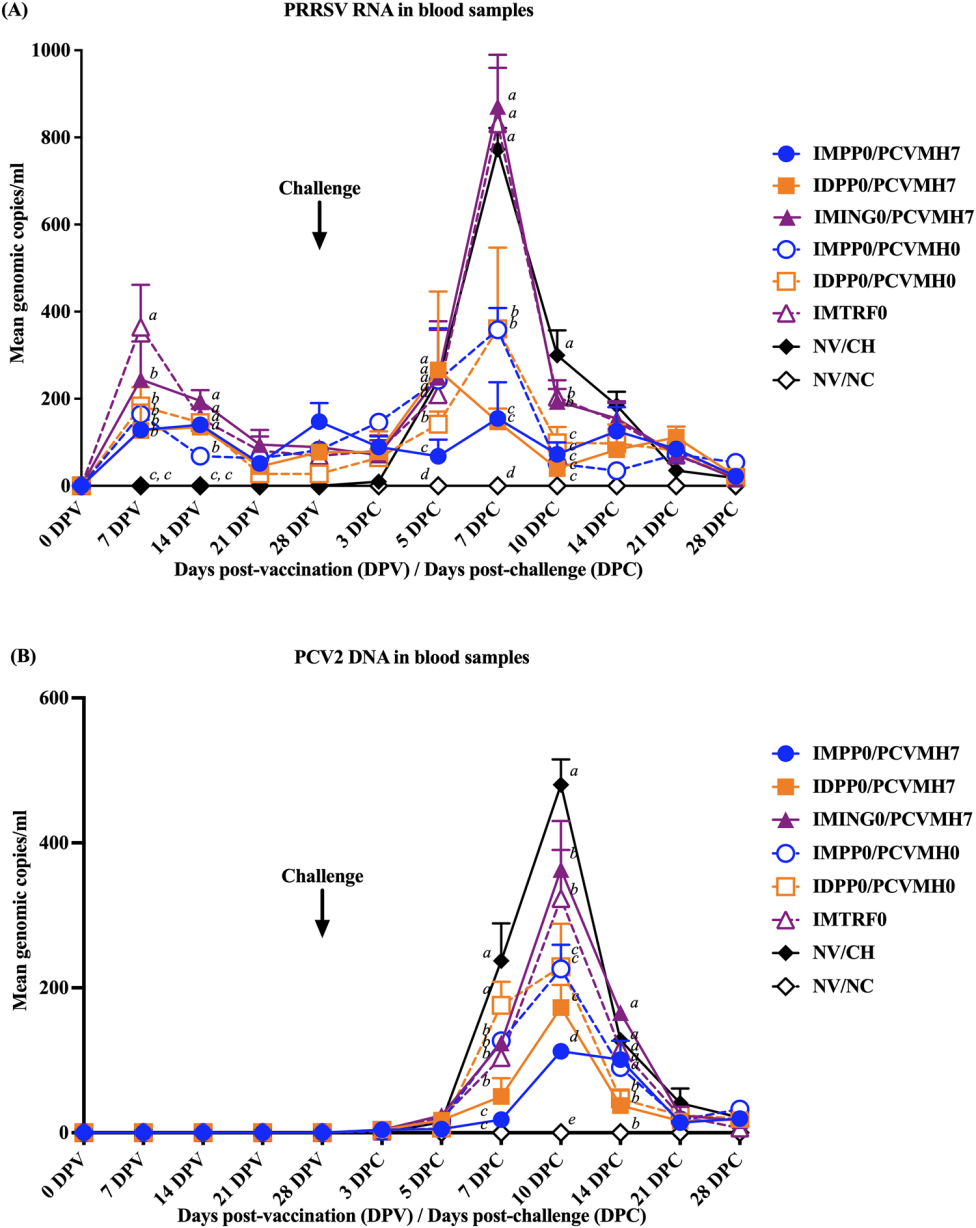
Mean genomic copies of (**A**) PRRSV RNA and (**B**) PCV2 DNA in the blood samples. Values expressed as mean ± SEM. The results were compared using two-way ANOVA for multiple comparisons. Different lowercase letters (a–c) indicate significant differences between treatment groups (*p* < 0.05) for each time-point.

No PRRSV RNA was detected in the blood sample of the NV/NC group throughout the experiment. Regardless of vaccine use, route, and timing of administration, PRRSV RNA in the blood of all vaccinated groups was first detected at a higher level at 7 DPV. Then PRRSV RNA continually decreased to the basal levels from 14 to 28 DPV (Fig. 5A). At 7 DPC, the IMTRF0 group a had significantly (*p* < 0.05) higher PRRSV RNA at 364.1 ± 97.2 copies/ml than those in the other vaccinated groups. At 14 DPV, the IMPP0/PCVMH0 group had a significantly (*p* < 0.05) lower PRRSV RNA level (68.55 ± 13.6 copies/ml) compared to those in the other vaccinated groups. There was no statistical difference in the PRRSV RNA levels between groups from 21 to 28 DPV.

Following challenge, the PRRSV RNA in the blood rapidly increased and reached peaks at 5 and 7 DPC. Then, the PRRSV RNA levels rapidly decreased to the basal levels from 10 to 28 DPC. There was no difference in PRRSV RNA levels among challenged groups at 3 DPC but an increase in PRRSV RNA levels was detected in all challenged groups at 5 DPC. At 5 DPC, the IMPP0/PCVMH7 group had a significantly (*p* < 0.05) lower PRRSV RNA (67.8 ± 18.3 copies/ml) than those in the other challenged groups. The reduction of PRRSV RNA in the blood was markedly observed among vaccinated groups from 7 to 10 DPC. At 7 DPC, the IMING0/PCVMH7 and IMTRF0 groups had significantly (*p* < 0.05) higher PRRSV RNA values (871.1 ± 118.2 and 831.2 ± 128.8 copies/ml) than that of the other vaccinated groups. In contrast, pigs in the IMPP0/PCVMH7 and IDPP0/PCVMH7 groups (154.6 ± 83.1 and 147.1 ± 30.3 copies/ml) had significantly (*p* < 0.05) lower PRRSV RNA values than those in the other groups at 7 DPC. At 10 DPC, pigs in the IMING0/PCVMH7 and IMTRF0 groups had significantly (*p* < 0.05) higher PRRSV RNA values (194.1 ± 28.3 and 204.1 ± 38.8 copies/ml) than that of the other vaccinated groups. While there was no statistical difference in the PRRSV RNA values between 14 and 28 DPC (Fig. 5A).

### Quantification of PCV2 DNA in blood

No PCV2 DNA detected in the blood sample of the NV/NC group throughout the experiment and there was no PCV2 DNA in the blood sample from any of pigs following vaccination (Fig. 5B). Following challenge, PCV2 DNA in all challenged groups rapidly increased and reached peaks at 10 DPC then gradually decreased to the basal levels from 14 to 28 DPC. PCV2 DNA among challenged groups was not different at 3 and 5 DPC. The IDPP0/PCVMH0 and NV/CH groups had significantly (*p* < 0.05) higher PCV2 DNA values (175.9 ± 32.4 and 237.4 ± 51.4 copies/ml) than those in the other challenged groups at 7 DPC. Meanwhile, pigs in the IMPP0/PCVMH7 group had significantly (*p* < 0.05) lower levels of PCV2 DNA (18.2 ± 2.2 and 112.3 ± 8.9 copies/ml) than that of the other challenged groups at 7 and 10 DPC. Pigs in the IMING0/PCVMH7 and IMTRF0 groups had significantly higher PCV2 DNA values (363.2 ± 67.1 and 323.2 ± 54.2 copies/ml) than that of the other vaccinated groups at 10 DPC. At 14 DPC, pigs in the IDPP0/PCVMH7 and IDPP0/PCVMH0 groups had significantly (*p* < 0.05) lower PCV2 DNA values (37.5 ± 21.5 and 46.9 ± 12.9 copies/ml) than that of the other challenged groups. There was no statistical difference in the PCV2 DNA among groups from 21 to 28 DPC (Fig. 5B).

### Macroscopic and microscopic lung lesions

Macroscopic and microscopic lung lesions are summarized in Table 2. The macroscopic lung lesions induced by PRRSV were characterized by multifocal, tan-molted areas with irregular and indistinct borders. The reduction of lung lesions was observed in all vaccinated groups regardless of the vaccine use and time of administration, except for the IMING0/PCVMH7 and IMTRF0 groups. At 7 DPC, pigs in the IMING0/PCVMH7 and IMTRF0 groups showed no difference in macroscopic lung lesion scores compared with the NV/CH group and had significantly (*p* < 0.05) higher macroscopic lung lesion scores of 63.0 ± 12.5 and 65.0 ± 13.5 than those in the other vaccinated-, challenged groups. In addition, pigs in the IMPP0/PCVMH7 group had a significantly (*p* < 0.05) lower macroscopic lung lesion score (23.6 ± 13.5) than that of the other challenged groups at 7 DPV.

**Table 2:** The mean values of macroscopic-, microscopic lung scores, and antigen scores in tissues at 7 DPC.

	IMPP0/PCVMH7	IDPP0/PCVMH7	IMING0/PCVMH7	IMPP0/PCVMH0	IDPP0/PCVMH0	IMTRF0	NV/CH	NV/NC
Macroscopic lung scores	23.6 ± 13.5^c^	36.3 ± 12.6^b^	63.0 ± 12.5^a^	40.3 ± 5.1^b^	38.3 ± 3.3^b^	65.0 ± 13.5^a^	57.6 ± 11.6^a^	0.00 ± 0.00^d^
Microscopic lung scores	1.15 ± 0.16^c^	1.33 ± 0.16^b^	1.63 ± 0.22^b^	1.61 ± 0.17^b^	1.56 ± 0.17^b^	2.23 ± 0.12^a^	2.30 ± 0.10^a^	0.00 ± 0.00^d^
PRRSV antigen scores	3.6 ± 0.5^c^	4.3 ± 0.6^c^	9.2 ± 0.3^b^	4.3 ± 0.1^c^	5.2 ± 0.3^c^	9.8 ± 0.5^b^	15.6 ± 1.6^a^	0.00 ± 0.00^d^
PCV2 antigen scores	0.82 ± 0.06^c^	0.93 ± 0.16^c^	1.33 ± 0.32^b^	1.05 ± 0.42^c^	1.06 ± 0.17^c^	1.53 ± 0.32^b^	3.30 ± 0.20^a^	0.00 ± 0.00^d^

Values expressed as mean ± SEM. The results were compared using two-way ANOVA for multiple comparisons.

Different lowercase letters (a–d) indicate significant differences between treatment groups (*p* < 0.05) for each day.

For microscopic lung lesion scores, the lesions were characterized by thickened septa with an increased number of interstitial macrophages and lymphocytes and by type II pneumocyte hyperplasia. Like macroscopic lung lesion scores, pigs in the IMING0/PCVMH7 and IMTF0 groups (1.63 ± 0.22 and 2.33 ± 0.12) had significantly (*p* < 0.05) microscopic lung lesion scores than in the IDPP0/PCVMH7 and IDPP0/PCVMH0 groups (1.33 ± 0.16 and 1.36 ± 0.17). While the microscopic lung lesion score of pigs in the IMPP0/PCVMH7 group was the lowest (1.15 ± 0.16) compared to that of the other groups (Table 2).

### Immunohistochemistry (IHC)

The mean value of PRRSV- and PCV2-specific antigen scores in tissues are summarized in Table 2. Pigs in the NV/CH group had significantly (*p* < 0.05) higher PRRSV- and PCV2 antigen scores (15.6 ± 1.6 and 3.30 ± 0.2) than those in the other challenged groups. In accordance with the macroscopic and microscopic lung lesion scores, the Prime Pac vaccination groups showed significantly lower antigen scores than that of the Ingelvac vaccinated groups regardless of the vaccine use and time of administration. For PRRSV antigen score, pigs in the IMPP0/PCVMH7, IDPP0/PCVMH7, IMPP0/PCVMH0 and IDPP0/PCVMH0 groups had significantly (*p* < 0.05) lower antigen scores of 3.6 ± 0.5, 4.3 ± 0.6, 4.3 ± 0.1 and 5.2 ± 0.3 than that in the IMING0/PCVMH7 and IMTRF0 groups (9.2 ± 0.3 and 9.8 ± 0.5) at 7 DPC. In addition to PRRSV antigen score, pigs in the IMING0/PCVMH7, IDPP0/PCVMH7, IMING0/PCVMH0 and IDPP0/PCVMH0 groups had significantly (*p* < 0.05) lower PCV2 antigen scores of 0.82 ± 0.06, 0.93 ± 0.16 and 1.05 ± 0.42 and 1.06 ± 0.17 than that in the IMING0/PCVMH7 (1.33 ± 0.32), and IMTRF0 groups (1.53 ± 0.32).

## Discussion

Analogous to the vaccination of juvenile swine at weaning, vaccination is commonly practiced in pediatric immunization programs around the world. Children may be vaccinated with different antigens in different limbs simultaneously and this is known as concurrent vaccination. Concurrent vaccination with different antigens has been demonstrated to lead to suboptimal immune responses in some pediatric settings^34–36^. This has not been reliably demonstrated in swine vaccination and it is highly likely that such interactions are likely to be vaccine component dependent. Therefore, it is critical to evaluate all possible combinations individually, ideally in field studies to understand the immune responses. The present study investigated the immune response of concurrent vaccination with different modified-live PRRSV-2 vaccines, PCV2a-based, and *Mycoplasma (M.) hyopneumoneae* subunit vaccines when administrated via intramuscular or intradermal routes. In addition to the immune response, the protective efficacy against co-challenge with HP-PRRSV-2 and PCV2 was also evaluated.

Increasing anti-PRRSV antibody responses detected by ELISA from 7 to 14 days post-vaccination are a common feature of PRRSV MLV immunization^24,48^, and in most groups, challenge had a boosting effect on their production. Administration route does not seem to influence this response. Even though ELISA-detected antibodies are not correlated with PRRSV protection (contrarily to viral neutralizing antibodies), it is remarkable that challenge did not result in a boosted antibody response in the IMTRF0 group, which is the one with the most severe lung lesion scores at 7 days post-challenge. In this group, ELISA-detected anti-PRRSV antibody titers were significantly lower than in the other vaccinated groups. This might point to a limited ability of the corresponding vaccine at triggering an appropriate Th2 response in pigs. Sero-neutralizing titers are not significantly lower in this group (data not shown), which further documents that Th2 response measured by ELISA is not a correlate to protection.

All piglets included in the study presented detectable anti-PCV2 antibodies on the day of vaccination. The kinetics observed in the NV/NC group are concordant with the presence of maternally derived antibodies (MDA) decaying over the next 4 weeks. Conventional piglets were used in the present trials and nearly all sow farms in Thailand use quarterly mass-vaccination protocols. Thus, the presence of MDA at the start of the study was to be expected. A significant increase in ELISA antibodies titers was observed on 7 DPV in one vaccinated group, being administered on the same single day: IDPP0/PCVMH0. This increase was transient, but the boosting effect of PCV2 challenge was also significant in this group. In two other vaccinated groups (IDPP0/PCVMH7 and IMTRF0), the boosting effect of PCV2 challenge was more limited and delayed to weeks post-inoculation, without obvious association with PCV2 IHC scores. These two groups were those with the lowest MDA levels at challenge, but whether this is an incidental observation or has a causal link with antibody response would need further exploration.

In all vaccination groups, immunization was followed by a limited viraemia, as is classically described for PRRSV MLV vaccines^53,54^. Post immunization viremia levels were broadly similar across vaccines and administration routes across the 4-week post vaccination period. Simultaneous challenge with an HP-PRRSV-2 strain and PCV2 of groups of pigs with different vaccination protocols/administration routes shows that all groups (except the non-vaccinated) develop comparable viraemia profiles, as well as specific antibody responses. However, in most cases, the ID administration route, irrespective of the vaccine brand, provides improved post-challenge growth performance and significantly decreased lung histopathological scores. Also, ID vaccination seems to induce an earlier development and a significantly higher level of activation of the IFN-γ-SC, both against homologous and heterologous HP-PRRSV-2 viruses. The IMPP0/PCVMH0 group showed a relatively lower ADWG over the 4 weeks post-vaccination period compared to other groups (data not shown). However, this was not reflected in the immune responses or the viremia levels of either PCV2 or PRRSV, suggesting that other factors may have contributed to the slower growth rate in this group. This confirms that all commercial vaccines are well-tolerated by the pigs and have no deleterious impact on growth, irrespective of their administration route. While most challenge trials with PRRSV and PCV2 perform successive inoculations, several days apart, concomitant infection by both viruses has been shown to act synergistically in SPF piglets, inducing an excessive activation of Toll-like receptors signaling, possibly setting the stage for increased clinical severity of secondary infections^55^. The simultaneous co-infection challenge also matches more closely the field situation, where both viruses may be contemporaneously circulating^22,56^. The PCV2d strain used in the coinfection inoculum is a field strain from 2011 to 2012 outbreaks of PCVADs. In Thailand, the oldest presence of genotype PCV2d has been retrospectively established, and this genogroup became dominant in the Thai swine industry in 2013–2014^47^. Using PCV2d as a challenge strain thus corresponds to the field situation.

In a recent study exploring the protective effect of Ingelvac PRRS MLV in high-health pigs challenged with PRRSV-2 and PCV2, no significant growth differences were observed over the 42 days post-challenge between vaccinated and non-vaccinated groups^57^. No double negative (non-vaccinated and non-challenged) group was included in that study, however. Also, the PRRSV-2 strain used in the present experiment is a highly pathogenic strain^25,48^. In an experiment designed to assess the pathogenicity of a French PRRSV-1 field strain derived from a modified-live vaccine, simultaneous inoculation of PCV2b and either PRRSV-1 strain (either the field or the MLV strain) has been performed in unvaccinated SPF pigs^58^. The group of pigs coinfected with the field PRRSV strain and PCV2 developed more severe lung lesions and produced a higher transmission rate to naïve contacts, than the group coinfected by the MLV and PCV2, but no difference in ADWG was measured between groups, possibly owing to the high health status of the animals and the limited virulence of the PRRSV strain used. In the present results, whether vaccines were injected on 0 DPV or on 0 and 7 DPV, groups that were vaccinated through the ID route presented with a relatively higher or similar ADWG, depending on which IM vaccine they were compared to. This is in line with lung lesion scoring, both at the macroscopic and microscopic scales, where the one group whose score was not statistically different from the NV/NC group were IM-vaccinated (IMTRF0).

No mortality was observed after challenge, as previously observed in MLV-vaccinated pigs challenged with the same HP-PRRSV-2 strain, but without PCV2^48^. Two groups of pigs developed severe hyperthermia after challenge: IMING0/PCVMH7 and IMTRF0, both being groups of pigs vaccinated by the IM route. In the first group, fever occurrence was comparable to that of the NV/CH group in amplitude and duration. In all other MLV-vaccinated groups, no significant change in rectal temperature was observed, compared to the NV/NC group. In a recent trial, high-health pigs vaccinated with Ingelvac PRRS MLV have been inoculated with a PRRSV-2 and a PCV2b US strains 4 weeks post-vaccination^57^, producing increased PCVAD clinical signs and pathology 3 weeks after coinfection (and reduced PRRS clinical signs over the three first week post-challenge). That trial did not include PCV2 vaccinated pigs, and in the present experiments, pigs were sacrificed on 7 DPC, precluding any further observation. Also, the enhanced virulence of a PRRSV MLV-1-like strain by co-infection with PCV2 that has recently been described^58^ was not observed in the present experiment. The fact that conventional piglets (rather than SPF) were used might provide an explanation for this difference.

In the non-vaccinated group, challenge induced a PRRSV viraemia peak at 7 days PI, while NV/NC pigs remained virus-free. Two groups (IMING0/PCVMH7 and IMTRF0) developed a viraemia of a comparable amplitude and duration to that of the NV/CH group. However, these groups presented with significantly different histopathological and IHC lung scores. In the IMING0/PCVMH7 group, both scores were significantly lower than those measured in the NV/CH group. This might point to a control of the challenge virus in the lungs—and hence to a more limited inflammatory reaction induced by the HP-PRRSV-2 strain^48,59^ than in the NV/CH group, in relation to previous immunization. On the other hand, in the IMTRF0 group, histopathological and IHC lung scores were not significantly different than those in the NV/CH group, which points of a lack of protection towards the heterologous HP-PRRSV-2 challenge strain. Whether or not these observations are associated with the fact that both these groups were those with the highest post-vaccination viraemia is not clear and might deserve further attention. All other vaccination groups had a comparable PRRSV viraemia profile post-challenge, with a transiently increased viraemia, limited to the 5–7 DPC—which points to a successful control of the challenge strain.

A strong PCV2 viraemia was observed in the NV/CH group, while none of the NV/NC animals had detectable PCV2 DNA in their serum, pointing to the absence of accidental natural infection during the trial. None of the vaccinated groups presented a comparable viraemia peak; the IMING0/PCVMH7 and IMTRF0 groups were those with the highest viremic peak among vaccinated groups. Albeit significantly lower than that observed in control animals, it was still significantly higher than that of all other groups (which had a common viraemia pattern). In both IMING0/PCVMH7 and IMTRF0 groups, the IHC lung lesion scores were significantly lower than those of the NV/CH pigs which confirms that the corresponding vaccines provide significant protection against PCV2. Since all commercial PCV2 vaccines included in this trial were designed using a PCV2a stain, the fact that PCV2d might partially escape cross-protection from PCV2a-based vaccines^14^ is not relevant to the present study. More probably, co-infection might have triggered PCV2 replication in some pigs or pig groups, as has been previously observed by others^57,60^.

Several vaccination route specific differences were observed. These were in the induction of the IL-10 secretion and IFN-γ-SC induction. ID-vaccinated pigs had significantly lower IL-10 levels and higher IFN-γ-SC frequencies compared to that of the IM-vaccinated pigs regardless of the vaccine use and timing of administration. Regarding cell-mediated immune response (Th1), both the IM and the ID routes are acknowledged to induce specific IFN-γ-SC following MLV injection, which helps reduce PRRSV viraemia^61^. In the present trials, when stimulated with the homologous vaccine strains, IFN-γ-SC were detectable in the serum of all vaccinated groups as early as 3 weeks post-immunization. This was also true for IFN-γ-SC induced by heterogenous (HP-PRRSV-2) stimulation, which agrees with previous experiments^48,54^. Once pigs were challenged with the PRRSV-2 strain, a comparable pattern of continued induction of “homologous” cells was observed in all vaccinated groups. However, the amplitude of the response was significantly stronger in groups that had received the MLV vaccine by the ID route (i.e., IDPP0/PCVMH7 and IDPP0/PCVMH0), which is in line with the recent description of the positive impact of ID over IM routes^62^. This difference disappeared for “heterologous” cells, which were highly induced after challenge in all groups, with the notable exception of the IMTRF0 group. Comparable to others, although with different immunization products, both injection routes provided good protection against the heterologous challenge, while they produced different levels of IFN-γ-SC^62^. Induction of IFN-γ-SC was also observed in the 2 weeks following PCV2 vaccination, upon in vitro stimulation of PBMCs with the heterologous PCV2d strain (all vaccines are based on a PCV2a capsid structure). The PCV2d challenge allowed the induction of such cells to peak at 7 DPC, with a remarkable increase in the IDPP0/PCVMH7 group, well-above the peak obtained in the other groups, and a surprising absence of peak in the IMTRF0 group. In the latter group, this relates to the limited control of PCV2 peak viraemia after challenge. The absence of response in the NV/CH group may by contrast suggest that vaccination in the face of maternal antibodies in the study did induce IFN-γ-SC.

Although all vaccines tested in the present experiments induced a comparable pattern of increase in IL-10 concentration upon PBMC stimulation with homologous strains, there was a difference in the peak of these responses between vaccines, a difference that had previously been highlighted by others in previous studies^63^. In the present results, there seems to be a delay in the IL-10 peak, occurring at 7 DPV in the groups vaccinated by the IM route and at 14 DPV in those vaccinated by the ID route. In comparable immunization protocols (although with different PRRS MLVs), the IL-10 peak was observed at 14 DPV^25^ and 21 DPV^48^ for both injection routes. It is possible that each MLV strain or even each vaccine formulation has different abilities in triggering effectors of IL-10 secretion. One interesting point is that the IMTRF0 is the group with the highest IL-10 peak. IL-10 being an immunosuppressive cytokine, it suggests that this vaccine might induce a higher IL-10 response and possibly induce an earlier or stronger Treg activation (not measured), explaining the lower levels in the other markers of the adaptive immune response measures in the present experiments. In any case, it illustrates that the context of presentation of the MLV may be modulated by adjuvants, since the vaccine used in the IMTRF0 group combines in a single product for single administration a PRRSV live strain and two inactivated valences (PCV2 and *M. hyopneumoniae*). Overall, these experiments demonstrate that commercially available multivalent vaccines, with single or two-injection priming protocols, induce protection upon concomitant challenge with HP-PRRSV-2 and PCV2. The ID immunization route provides an improved specific immune response, both through Th1- and Th2-effectors, over the IM route. The limited ability of the MLV used in the IMTRF0 group to mount immunity markers and lesion scores at comparable levels to the other PRRSV MLVs might deserve further investigation, through the exploration of Treg activation pathway.

Our findings demonstrate that when comparing similar vaccines administered through similar routes with the only difference concurrently or separately, concurrent vaccination of PRRSV and PCV2 antigens was well-tolerated without adverse reactions. Differences in the immune responses were observed mainly due to the vaccination route. The ID vaccination had significantly lower IL-10 levels and higher IFN-γ-SC values when compared to the IM-vaccinated groups. In terms of clinical outcomes, only one group (IMPP0/PCVMH7) showed statistically better efficacy compared to its concurrent counterpart, as demonstrated by lower viremia levels, lung lesions, and antigen scores. However, these consequences only appeared in the IM route. When compared to the ID routes, these outcomes disappeared.

In terms of clinical relevance, our study confirms that concurrent vaccination of PRRSV-2 MLV and PCV2 via either the IM or the ID routes could be applicable to assist with the control of PRDC without adding additional reactogenicity. Therefore, producers can select concurrent or separate vaccination depending on the situation on the farm. In vertically integrated operations with multi-site production where lesser health challenges exist, the consumer has higher visibility of production, and meat quality and animal welfare improvements are most important. Concurrent vaccination of PRRSV and PCV2 vaccines via the ID route might be a useful alternative tool for producers wishing to reduce the handling of pigs and hence acute stress while simultaneously controlling PRRSV and PCV2 infections. In this circumstance, intradermal vaccination has several additional benefits, ranging from improved food safety, due to the elimination of needles, to potential biosecurity improvements from the reduction in iatrogenic disease transmission^43^. In situations where the ID vaccination is not possible due to a lack of equipment, the IM vaccination may be the only option available. Based on our findings, if applying IM vaccination, better efficacy results are obtained by splitting up the antigens compared with concurrent vaccination.

Our study contained several limitations. Firstly, it was performed in conventional pigs, owing to the objectives of providing results useful to practitioners and farmers, especially in controlling PRRSV. This might have hampered a clear-cut picture of the adaptive immune response of piglets with MDA against PCV2, but again, this reflects field conditions. Secondly, our common inoculation of both an HP-PRRSV-2 strain and PCV2 might have been seen as an excessive challenge to swine health, but it was approved by the university’s ethical committee and reflects a common situation in the field. Also, the fact that no mortality was observed, including in the NV/NC group, which supports our choice. We did not include a *Mycoplasma hyopneumoniae* challenge in this study because owing to epidemiological reasons, exposure to this pathogen usually occurs later in production^28^. Future studies will add this challenge while maintaining the pre-existing challenge model. Thirdly, it might appear that the delay between challenge and termination of the study (7 days) is too short to see a clinical outcome of the PCV2 challenge. This limitation is substantiated by the observation of PCVAD signs in pigs at 4 weeks post-challenge in another experimental double challenge^57^ and by the high IHC PCV2 score observed in the NV/CH group. However, our PCV2 DNA in the blood samples out of 28 DPC serves as an appropriate proxy.

## Conclusions

Based on the immune parameters assessed in the study, concurrent vaccination of both PCV2 and PRRSV antigens does not reduce vaccine efficacy or introduce additional reactogenicity. Pigs vaccinated by the concurrent ID route benefitted from equivalent or better immune responses and protection against the dual challenge than those vaccinated by the corresponding separate route. Meanwhile, the protection in the IM route appeared to be better in the corresponding to the separate route than in the concurrent route. The single group that received a trivalent mixed vaccine mounted a more limited response in immunity development and efficacy markers compared with all the other groups.

## Data Availability

The datasets used and/or analysed during the current study available from the corresponding author on reasonable request.

## References

[1] OIE. *The OIE terrestrial Animal Health Code*. https://www.oie.int/index.php?id=169&L=0&htmfile=chapitre_notification.htm Accessed 2 April 2021 (2019).

[2] Segalés J, Allan GM, Domingo M (2019). Diseases of Swine.

[3] Meng XJ (2012). Emerging and re-emerging swine viruses. Transbound. Emerg. Dis..

[4] Zimmerman JJ (2019). Diseases of Swine.

[5] Balka G (2018). Genetic diversity of PRRSV 1 in Central Eastern Europe in 1994–2014: Origin and evolution of the virus in the region. Sci. Rep..

[6] Le Gall A (1998). Molecular variation in the nucleoprotein gene (ORF7) of the porcine reproductive and respiratory syndrome virus (PRRSV). Virus Res..

[7] Nelsen CJ, Murtaugh MP, Faaberg KS (1999). Porcine reproductive and respiratory syndrome virus comparison: Divergent evolution on two continents. J. Virol..

[8] Kim HK (2009). Genetic analysis of ORF5 of recent Korean porcine reproductive and respiratory syndrome viruses (PRRSVs) in viremic sera collected from MLV-vaccinating or non-vaccinating farms. J. Vet. Sci..

[9] Stadejek T, Stankevicius A, Murtaugh MP, Oleksiewicz MB (2013). Molecular evolution of PRRSV in Europe: Current state of play. Vet. Microbiol..

[10] Zhou L, Yang H (2010). Porcine reproductive and respiratory syndrome in China. Virus Res..

[11] Gillespie J, Opriessnig T, Meng XJ, Pelzer K, Buechner-Maxwell V (2009). Porcine circovirus type 2 and porcine circovirus-associated disease. J. Vet. Intern. Med..

[12] Pallares FJ (2002). Porcine circovirus type 2 (PCV-2) coinfections in US field cases of postweaning multisystemic wasting syndrome (PMWS). J. Vet. Diagn. Investig..

[13] Opriessnig T, Gimenez-Lirola LG, Halbur PG (2011). Polymicrobial respiratory disease in pigs. Anim. Health Res. Rev..

[14] Bandrick M (2020). T cell epitope content comparison (EpiCC) analysis demonstrates a bivalent PCV2 vaccine has greater T cell epitope overlap with field strains than monovalent PCV2 vaccines. Vet. Immunol. Immunopathol..

[15] Neumann EJ (2005). Assessment of the economic impact of porcine reproductive and respiratory syndrome on swine production in the United States. J. Am. Vet. Med. Assoc..

[16] Arruda AG (2017). Investigation of the occurrence of porcine reproductive and respiratory virus in swine herds participating in an area regional control and elimination project in Ontario, Canada. Transbound. Emerg. Dis..

[17] Montaner-Tarbes S, Del Portillo HA, Montoya M, Fraile L (2019). Key gaps in the knowledge of the porcine respiratory reproductive syndrome virus (PRRSV). Front. Vet. Sci..

[18] Chase-Topping M (2020). New insights about vaccine effectiveness: Impact of attenuated PRRS-strain vaccination on heterologous strain transmission. Vaccine.

[19] Chae C (2021). Commercial PRRS modified-live virus vaccines. Vaccines..

[20] Renukaradhya GJ, Meng XJ, Calvert JG, Roof M, Lager KM (2015). Live porcine reproductive and respiratory syndrome virus vaccines: Current status and future direction. Vaccine.

[21] Dee S, Otake S, Deen J (2010). Use of a production region model to assess the efficacy of various air filtration systems for preventing airborne transmission of porcine reproductive and respiratory syndrome virus and *Mycoplasma hyopneumoniae*: Results from a 2-year study. Virus Res..

[22] Saade G (2020). Coinfections and their molecular consequences in the porcine respiratory tract. Vet. Res..

[23] Yin SH (2013). Concurrent porcine circovirus type 2a (PCV2a) or PCV2b infection increases the rate of amino acid mutations of porcine reproductive and respiratory syndrome virus (PRRSV) during serial passages in pigs. Virus Res..

[24] Madapong A (2017). Humoral immune responses and viral shedding following vaccination with modified live porcine reproductive and respiratory syndrome virus vaccines. Arch. Virol..

[25] Madapong A, Saeng-Chuto K, Boonsoongnern A, Tantituvanont A, Nilubol D (2020). Cell-mediated immune response and protective efficacy of porcine reproductive and respiratory syndrome virus modified-live vaccines against co-challenge with PRRSV-1 and PRRSV-2. Sci. Rep..

[26] Martínez-Miró S (2016). Causes, consequences and biomarkers of stress in swine: An update. BMC Vet. Res..

[27] Gimsa U, Tuchscherer M, Kanitz E (2018). Psychosocial stress and immunity—What can we learn from pig studies?. Front. Behav. Neurosci..

[28] Maes D (2008). Control of *Mycoplasma hyopneumoniae* infections in pigs. Vet. Microbiol..

[29] Rose N, Opriessnig T, Grasland B, Jestin A (2012). Epidemiology and transmission of porcine circovirus type 2 (PCV2). Virus Res..

[30] Nilubol D, Tripipat T, Hoonsuwan T, Tipsombatboon P, Piriyapongsa J (2013). Genetic diversity of the ORF5 gene of porcine reproductive and respiratory syndrome virus (PRRSV) genotypes I and II in Thailand. Arch. Virol..

[31] Martelli P (2013). Concurrent vaccinations against PCV2 and PRRSV: Study on the specific immunity and clinical protection in naturally infected pigs. Vet. Microbiol..

[32] Park C, Oh Y, Seo HW, Han K, Chae C (2013). Comparative effects of vaccination against porcine circovirus type 2 (PCV2) and porcine reproductive and respiratory syndrome virus (PRRSV) in a PCV2-PRRSV challenge model. Clin. Vaccine Immunol..

[33] Li Z (2016). Pathological and immunological characteristics of piglets infected experimentally with a HP-PRRSV TJ strain. BMC Vet. Res..

[34] Dagan R, Eskola J, Leclerc C, Leroy O (1998). Reduced response to multiple vaccines sharing common protein epitopes that are administered simultaneously to infants. Infect. Immun..

[35] Kitchin NR (2007). Evaluation of a diphtheria-tetanus-acellular pertussis-inactivated poliovirus-*Haemophilus influenzae* type b vaccine given concurrently with meningococcal group C conjugate vaccine at 2, 3 and 4 months of age. Arch. Dis. Child..

[36] Burrage M (2002). Effect of vaccination with carrier protein on response to meningococcal C conjugate vaccines and value of different immunoassays as predictors of protection. Infect. Immun..

[37] Chase C, Daniels C, Garcia R, Milward F (2008). Needle-free injection technology in swine: Progress toward vaccine efficacy and pork quality. J. Swine Health Prod..

[38] Fu TM (1997). Priming of cytotoxic T lymphocytes by DNA vaccines: Requirement for professional antigen presenting cells and evidence for antigen transfer from myocytes. Mol. Med..

[39] Ferrari L (2013). Lymphocyte activation as cytokine gene expression and secretion is related to the porcine reproductive and respiratory syndrome virus (PRRSV) isolate after in vitro homologous and heterologous recall of peripheral blood mononuclear cells (PBMC) from pigs vaccinated and exposed to natural infection. Vet. Immunol. Immunopathol..

[40] Martelli P (2014). Systemic and local immune response in pigs intradermally and intramuscularly injected with inactivated *Mycoplasma hyopneumoniae* vaccines. Vet. Microbiol..

[41] Sno M (2016). Efficacy and safety of a new intradermal PCV2 vaccine in pigs. Trials Vaccinol..

[42] Imeah B, Penz E, Rana M, Trask C, Needle-less Injector Study Team (2020). Economic analysis of new workplace technology including productivity and injury: The case of needle-less injection in swine. PLoS ONE.

[43] Madapong A, Saeng-Chuto K, Tantituvanont A, Nilubol D (2021). Safety of PRRSV-2 MLV vaccines administrated via the intramuscular or intradermal route and evaluation of PRRSV transmission upon needle-free and needle delivery. Sci. Rep..

[44] Halbur PG (1995). Comparison of the pathogenicity of two US porcine reproductive and respiratory syndrome virus isolates with that of the Lelystad virus. Vet. Pathol..

[45] Shi M (2010). Phylogeny-based evolutionary, demographical, and geographical dissection of North American type 2 porcine reproductive and respiratory syndrome viruses. J. Virol..

[46] Nilubol D, Tripipat T, Hoonsuwan T, Kortheerakul K (2012). Porcine reproductive and respiratory syndrome virus, Thailand, 2010–2011. Emerg. Infect. Dis..

[47] Thangthamniyom N (2017). Genetic diversity of porcine circovirus type 2 (PCV2) in Thailand during 2009–2015. Vet. Microbiol..

[48] Madapong A (2020). Immune response and protective efficacy of intramuscular and intradermal vaccination with porcine reproductive and respiratory syndrome virus 1 (PRRSV-1) modified live vaccine against highly pathogenic PRRSV-2 (HP-PRRSV-2) challenge, either alone or in combination with of PRRSV-1. Vet. Microbiol..

[49] Chaikhumwang P (2015). Dynamics and evolution of highly pathogenic porcine reproductive and respiratory syndrome virus following its introduction into a herd concurrently infected with both types 1 and 2. Infect. Genet. Evol..

[50] Opriessnig T (2004). Experimental reproduction of postweaning multisystemic wasting syndrome in pigs by dual infection with *Mycoplasma hyopneumoniae* and porcine circovirus type 2. Vet. Pathol..

[51] Halbur PG (1996). Comparative pathogenicity of nine US porcine reproductive and respiratory syndrome virus (PRRSV) isolates in a five-week-old cesarean-derived, colostrum-deprived pig model. J. Vet. Diagn. Investig..

[52] Park C, Seo HW, Han K, Kang I, Chae C (2014). Evaluation of the efficacy of a new modified live porcine reproductive and respiratory syndrome virus (PRRSV) vaccine (Fostera PRRS) against heterologous PRRSV challenge. Vet. Microbiol..

[53] Martinez-Lobo FJ (2013). Safety of porcine reproductive and respiratory syndrome modified live virus (MLV) vaccine strains in a young pig infection model. Vet. Res..

[54] Jeong J, Choi K, Kang I, Park C, Chae C (2016). Evaluation of a 20 year old porcine reproductive and respiratory syndrome (PRRS) modified live vaccine (Ingelvac((R)) PRRS MLV) against two recent type 2 PRRS virus isolates in South Korea. Vet. Microbiol..

[55] Dong VH (2015). Expression of Toll-like receptor signaling-related genes in pigs co-infected with porcine reproductive and respiratory syndrome virus and porcine circovirus type 2. Res. Vet. Sci..

[56] Ma Z (2021). Epidemiological investigation of porcine circovirus type 2 and its coinfection rate in Shandong province in China from 2015 to 2018. BMC Vet. Res..

[57] Niederwerder MC (2015). Vaccination with a porcine reproductive and respiratory syndrome (PRRS) modified live virus vaccine followed by challenge with PRRS virus and porcine circovirus type 2 (PCV2) protects against PRRS but enhances PCV2 replication and pathogenesis compared to results for nonvaccinated cochallenged controls. Clin. Vaccine Immunol..

[58] Eclercy J (2020). PCV2 co-infection does not impact PRRSV MLV1 safety but enhances virulence of a PRRSV MLV1-like strain in infected SPF pigs. Vet. Microbiol..

[59] Nedumpun T (2017). Interleukin-1 receptor antagonist: An early immunomodulatory cytokine induced by porcine reproductive and respiratory syndrome virus. J. Gen. Virol..

[60] Opriessnig T (2012). Effect of porcine circovirus type 2a or 2b on infection kinetics and pathogenicity of two genetically divergent strains of porcine reproductive and respiratory syndrome virus in the conventional pig model. Vet. Microbiol..

[61] Charerntantanakul W (2006). Immune responses and protection by vaccine and various vaccine adjuvant candidates to virulent porcine reproductive and respiratory syndrome virus. Vet. Immunol. Immunopathol..

[62] Park C (2021). Intradermal co-inoculation of codon pair deoptimization (CPD)-attenuated chimeric porcine reproductive and respiratory syndrome virus (PRRSV) with Toll like receptor (TLR) agonists enhanced the protective effects in pigs against heterologous challenge. Vet. Microbiol..

[63] Rahe MC, Murtaugh MP (2017). Mechanisms of adaptive immunity to porcine reproductive and respiratory syndrome virus. Viruses..

